# Non-human contributions to personality neuroscience: From fish through primates – a concluding editorial overview

**DOI:** 10.1017/pen.2024.1

**Published:** 2024-02-06

**Authors:** N. McNaughton, Y. V. Lages

**Affiliations:** 1 Department of Psychology, University of Otago, Dunedin, New Zealand; 2 Department of Psychology, Pontifical Catholic University of Rio de Janeiro, Rio de Janeiro, Brazil

**Keywords:** Evolution, personality, psychopathology, neuroscience, translation

## Abstract

This special issue attempts to integrate personality, psychopathology, and neuroscience as means to improve understanding of specific traits and trait structures in humans. The key strategy is to dive into comparative research using a range of species to provide simple models. This strategy has, as its foundation, the fact that the most basic functions, and their supporting neural systems, are highly conserved in evolution. The papers collected in the issue show that, from fish, through rats, to primates, the homologies in brain systems and underlying functions (despite species-specific forms of expression) allow simpler cases to provide insights into the neurobiology behind more complex ones including human. Our introductory editorial paper to this special issue took a bottom-up approach, starting with the genetics of conserved brain systems and working up to cognition. Here, we deconstruct the different aspects of personality, progressing from more complex ones in primates to least complex in fish. With the primate section, we summarize papers that discuss the factors that contribute to sociability in primates and how they apply to healthy and pathological human personality traits. In the rat section, the focus is driven by psychopathology and the way that “high” strains selected for extreme behaviors can illuminate the neurobiology of motivated responses to environmental cues. The section on fish summarizes papers that look into the most fundamental emotional reactions to the environment that are governed by primitive and conserved brain structures. This raises metatheoretical questions on the nature of traits and to a section that asks “which animals have personalities.” We believe that the issue as a whole provides a nuanced answer to this question and shines a new, comparative, light on the interpretation of personality structure and the effects on it of evolution.

This paper provides a concluding editorial overview of the smorgasbord of focussed reviews for our Special Issue, *Non-human contributions to personality neuroscience – from fish through primates*. As noted in our introductory editorial paper (Lages & McNaughton, 2022), the Special Issue aimed:

“…to make clear: (1) that non-human work of all types allows comparative analysis (from fish through primates) important for theories of personality in general and personality neuroscience in particular; (2) how strain derivation and neural manipulations generate non-human results that inform traits, particularly those of interest in human psychopathology (where Eysenck’s 3-factor model is still held in high regard, albeit with a need to rename his factors); (3) that observational non-human work, particularly in primates, can link to and inform the Big 5, HEXACO, etc; (4) that the different forms of non-human work can be naturally linked through study of the conserved brain systems involved – and so provide a basis for the integration of current hierarchical trait models of psychopathology (e.g., MMPI and HiTOP) with hierarchical trait models of healthy personality; (5) that, particularly between species, neural variation can help us link personality to brain systems. In sum, the Special Issue aims to show that, because of phylogenetic conservation of fundamental traits, even organisms as simple as fish can provide an architectural bedrock on which we can progressively build our understanding of the more elaborate superstructures on which personality depends in more complex organisms.”

In our editorial introduction, we argued that non-human models will help us understand neural (trait) sensitivities (Blanchard & Blanchard, 1989) and so sources of psychiatric illness (Greene et al., 2020; McNaughton, 2020) that are conserved by their adaptive consequences. The key conserved neural elements include diffuse ascending systems that provide global control of the human neocortex (Dubois et al., 2018, 2020), while having similar design and function from fish through primates, albeit acting on both older and more recently evolved structures in primates (Falcone et al., 2020; Miller et al., 2021). Our final step, then, will be to see what reduction to the basic “fish” story can tell us about the phylogenetic fundamentals of personality. But first, we will work our way down from greater to lesser encephalization, linking to human personality and psychopathology at each step.

## 1. Observation: personality in non-human primates

### 1.1. Observation versus self-report

It may seem that even our closest relatives, chimpanzees and bonobos, immediately present a problem for the assessment of personality homologies with humans. They cannot provide answers on self-report scales. However, the argument can be made that, particularly with rational rather than lexical construction, scales are often just proxies for reports that others would have made from sufficient observation of us. As argued by Colin DeYoung (personal communication, 13 February 2020):

“I think we have to be very careful not to assume that if trait measures assess behavioral tendencies, then these are merely ‘superficial patterns of verbal behavior’. Rather they are valid measures of behavioral tendencies more generally. I trust (because there is good validity evidence) that people who score high on extraversion do not merely say that they are more gregarious, assertive, talkative, dominant, excitable, enthusiastic, and joyful, but that they really are all of those things in their lives in general …. I hate the whole canard about the Big Five being ‘lexical’. That is totally irrelevant as long as they are measuring real, general patterns of behavior.”

With estimates of gregariousness, assertiveness, dominance, excitability, and enthusiasm, one might even think that the report of an observer could be more accurate than self-report. Importantly, these characteristics can clearly be assessed in humans and other social primates by observation of not only behavior in the more general sense but also facial expressions and facial morphology as social signals (§1.2). This allows extraction of factors such as dominance (§1.3) and extraversion (§1.4). Importantly, personality trait structures can be extracted from observations of primate social groups over time, and the resultant latent trait theory models show strong links to, for example, the human Big 5 model (Weiss, 2017; see also Weiss, 2022).

One would not expect facial expressions from fish, and it is quite hard to see a resemblance between those of a rat and a human. However, once we are dealing with primates, not only is the face itself more physically homologous with that of a human but also the muscular action patterns (Ekman, 1982) used to generate *functionally* similar emotional expressions are fundamentally the same (Redican, 1982). These action patterns are important from an ethological point of view since, with casual visual inspection, the expression may seem somewhat different (e.g., the play face homolog of the smile, see Parr & Waller, 2006; Redican, 1982, Figure 10.8, p. 254). This convergence extends also to the links between static facial features and traits, where newer non-human data are likely to help resolve older human discrepancies (§1.2).

The evolutionary homology of factors, such as dominance, seems clear despite the resultant personality structure being complicated by a shift in human societies from hierarchies towards egalitarianism (§1.3). Such homology also seems to extend to psychopathology with, for example, the primate homolog of extraversion, coupled with joint attention skills, and genetic and morphological data suggesting that primates can provide useful models of Autism Spectrum Disorder (§1.4). Primates, then, allow quite complex analysis of traits and trait structures and of their homology to humans based, fundamentally, on simple observation of natural characteristics including behavior.

### 1.2. Does the primate face cue personality?

This question is addressed by Wilson and Masilkova (2023), starting from the idea that *human* male facial morphology is linked to dominance with relative facial width supposedly linked to skull strength. Face width has been positively linked to dominance but with mixed and null findings. This leads to the argument:

“…expanding [analysis] to comparative work could help us understand which selection pressures might drive facial cues of dominance or other traits (Wilson, Weiss et al., 2020). … Personality, in particular Dominance-like traits [(see also §1.3)], seems to have links to facial metrics in all species studied, but these relationships depend on age, sex and even subspecies, suggesting divergent selection pressures driving facial cues to Dominance. Together, these findings paint an interesting but varied picture of the role of facial morphology in primate behaviour, suggesting that human studies have only just scratched the surface of a potentially much broader field.”

(Wilson & Masilkova, 2023, p. 3)

As a conclusion, the authors propose moving away from single measures, such as relative width and measures based on ratios, and approaching facial morphology in its multiple aspects and components, such as skill, flesh, and ornaments. Secondarily, the underlying bases of facial dysmorphism and its relation to personality must be better investigated, in its neuroendocrine, genetic, and developmental aspects (where primate data lag human). In addition to the inter-species perspective, the authors propose that translation might benefit from more encompassing human studies, with more diverse social-ecological samples. However, an additional issue is how far dominance reflects a single major factor in humans the way it does in other primate research.

### 1.3. Dominance and the evolution of primate sociality

Dominance in the simple instantaneous functional sense is clearly important for interactions in any social species. However, the presence and nature of one or more dominance traits needs to be established by observation of individuals over time. When this is done:

“…dominance traits are seen in virtually all primate species, and these dimensions reflect how adept an individual is at ascending within a social hierarchy. Among great apes, dominance is one of the most prominent personality factors but, in humans, dominance is usually modelled as a facet of extraversion. … Dominance itself can be subdivided into correlated subfactors: domineering, prestige, and leadership. Various explanations have been posed for why dominance has declined in prominence within human personality factor structures.”

(Altschul, 2024)

That primate dominance contains facets/subfactors with relatively high intercorrelations allows for homologies even when those facets become less intercorrelated:

“Weiss (2022) argues that the great ape dominance factors dissipated during human evolution and can now be found spread across different facets of different domains of the Big 5. Fearless dominance, which is a broad construct drawing on, in particular, facets of extraversion, neuroticism, and openness, is for this reason a good fit as a human analogue of great ape dominance. Fearless dominance converges with a general factor of dominance or power seeking, as well as any other general construct of dominance or assertiveness. Fearless dominance itself is strongly associated with low behavioural inhibition, high sensation and fun seeking, low anxiety and internalizing, and narcissistic personality disorder (NPD) diagnosis.”

(Altschul, 2024).

In apes, dominance is often critical for the climbing up to the leader position. However, Altschul points out that egalitarianism in early human cultures inverted this logic through subverted dominance, in which resource sharing and complying to social norms was encouraged. That said, the vast majority of current societies are shaped around hierarchy and displays of dominance may have been mitigated by language and an associated decrease in aggression as a means of resolving disputes. This would allow dominance to be dispersed among other personality constructs. Further, “the lexical method for deriving personality, i.e., using language and questions to tap into traits, is impacted by norms and morals inherent to the culture of the individual, and the language(s) of that culture” (Altschul, 2024).

It is possible, then, that our picture of the nature of human dominance is distorted by social and other display rules. However, dominance trait variation is also found among other primates:

“…consistent with a scenario in which the common ancestor of great apes and humans possessed a dominance factor that combined traits related to Fearless Dominance and Assertiveness, and where the covariation of these traits underwent rapid change during hominoid evolution. … [The] findings do not describe qualitative differences between humans and our great ape relations, or between great apes. Personality factors are, after all, models of correlations among traits. [The] findings are therefore better described as showing ranges of possibilities along a spectrum occupied by different species of primates, including our own.”

(Weiss, 2022, pp. 249–250).

Despite its more diverse nature in humans, Altschul (2024) emphasizes the importance of studying Dominance given:

“…[its] links to, for instance, age, sex, aggression, self-esteem, locus of control, stress, health, and multiple socioeconomic status indicators. …. Together, broad fearless dominance and narrower assertiveness aspects and facets raise a question of what it means for the ‘importance’ of a construct if it easily identifiable, measurable, and found across multiple levels, but is not obviously present at the level revealed by the most convenient statistical model. On the other hand, more recent developments in the ‘nuance’ oriented approach and causal modelling suggest that researchers ought to focus on the variables that are pertinent to their research question and think carefully about what covariates to include. In conclusion, dominance is a widespread, meaningful personality construct.”

### 1.4. Autism-related socio-communicative phenotypes

Hopkins et al. (2023) report data that combine assessment of communication bids (receptive joint attention, RJA, performance), personality (carer assessed), and gray matter volume across a range of brain areas (MRI) in chimpanzees:

“We found that, like humans, chimpanzees who performed worse on the RJA task had lower Extraversion scores. We also found that joint attention skills and several personality dimensions, including Extraversion, were significantly heritable. There was also a borderline significant genetic correlation between RJA and Extraversion. A conjunction analysis examining gray matter volume showed that there were five main brain regions associated with both higher levels of Extraversion and social cognition. These regions included the right posterior middle and superior temporal gyrus, bilateral inferior frontal gyrus, left inferior frontal sulcus, and left superior frontal sulcus, all regions within the social brain network.”

Hopkins et al. (2023, p. 1.)

They note that RJA is an important measure of the social reciprocal impairments that characterize autism spectrum disorder (ASD) and that “individuals with a diagnosis of, or at risk for, ASD … differ in personality … with lower Big 5 scores, particularly in Extraversion, Openness, Conscientiousness, Agreeableness and Emotional Stability” (Hopkins et al., 2023, p. 2). These parallels suggest that the comparison of low and high scoring chimpanzees can model social impairment and provide a platform for analyzing the underlying neural mechanisms, including for the social impairment in ASD.

## 2. Strain derivation – personality in rats

### 2.1. The rat strain-selection approach to traits

As we will also see with the fish work, the study of rat “personality” often uses a strain-separation approach. The key element of the logic involved is that an initial base population will show individual differences that can be inherited and so selected for. In principle, the resultant positive and negative selected strains represent extremes of the original, presumed dimensional, variation. These strains can be analyzed for behavioral, biochemical, and genetic differences. It is an important feature of such selection that even when carried out using very simple characters as the criterion, the resultant phenotypic changes can be quite broad because of the limited nature of the available sources of the variation on which selection can act (Broadhurst, 1957, 1975; Dugatkin, 2018; Trut, 1999).

In this section, we will compare results with three different selection criteria that focus on what can be viewed as different levels of defensive control and so different aspects of stress. These criteria were chosen on the basis of assumed homologies with human reactions This approach also provides a degree of overlap with major areas of the fish work and so the posing of direct comparative questions in the future. We will cover the resultant strain differences from an attempt to create a restricted model of trait anxiety (§2.2), through an attempt to create a broader model of internalizing and externalizing (§2.3), through to what appears to be a higher-order model of “emotionality” (§2.4). These can all be viewed as the derivation of trait models for which at least one extreme can reflect disorder or a disorder risk factor. We will finish with a more explicit (discovered rather than selected) rat model of attention deficit hyperactivity disorder – that nonetheless can also be seen as a model of trait variation in the human population and of its dopaminergic neurobiology.

### 2.2. The Carioca model of trait anxiety

Cruz et al. (2024) discuss the validation of the Carioca rat lines as models of low and high generalized anxiety traits. They started selecting the Carioca lines in 2008, breeding for high or low freezing in a contextual-conditional fear paradigm. They highlight the differences, here, of two forms of anxiety:

“State anxiety is a transient state of anxiety that occurs at a given moment in a specific context. It is an emotional state of anxiety that is directly related to the perception of a potential threat, such that its intensity tends to increase in the presence of the threat and ceases when it is no longer present. Trait anxiety refers to an individual’s vulnerability to express anxiety over time in different situations, a relatively stable component of personality that involves an intricate interplay between genetic and environmental factors for its expression.”

Cruz et al. (2024)

Bidirectional breeding reduces the chance of mistakenly interpreting a behavioral measure as reflecting trait anxiety when it results from:

“…[the] interplay between a trait anxiety component (which reflects an individual’s vulnerability or susceptibility to anxiety) and the situation that elicits state anxiety at the time of testing. Thus, when extreme forms of these traits are ignored, it is difficult, if not impossible, to dissociate adaptive defensive reactions from eventual maladaptive defensive reactions that are supposedly associated with specific anxiety disorders.”

Cruz et al. (2024)

The selective breeding resulted, as expected, in two lines: The Carioca contextual high- and low-freezing strains (CHF and CLF respectively). But (as in §2.3 and §2.4; see also Broadhurst, 1957, 1975; Dugatkin, 2018; Trut, 1999) the key question is what, other than the simple selection criterion, changed – and does it reflect a trait of interest. Cruz et al. (2024) ask how far the CHF rat line is a valid and reliable animal model of generalized anxiety disorder (GAD). Their Table 1 maps CHF rats to human generalized anxiety in terms of (their words): 1) High and diffuse anxiety in contextual fear conditioning, elevated plus maze, and avoidance behavior in the elevated T-maze; 2) Immobility/freezing behavior; 3) Different pattern of acquisition/extinction in response to context and cue; 4) Increased corticosterone serum levels; 5) Benzodiazepine and serotonergic anxiolytics attenuate freezing behavior in CHF but not CLF rats; and 6) Higher alcohol intake. CHF rats have also shown parallel changes in the function and structure of neural circuits thought to underlie anxiety (Dias et al., 2014; Lages et al., 2023; León et al., 2020).

In contrast, the low-freezing responses of CLF rats do not seem to be linked to lower anxiety, but rather to changes in their dopaminergic system – evidenced by altered responses to haloperidol and methylphenidate. Whether the CLF can represent an animal model of hyperactivity and attention disorders is being investigated (see also §2.5).

### 2.3. The Roman model of internalizing

Fernández-Teruel et al. (2023) also focused on avoidance learning but, more specifically, on its relation more to stress in general than anxiety in particular. The Roman high- and low-avoidance rat lines/strains (RHA/LHA, respectively) were bidirectionally selected for rapid (RHA) or extremely poor (RLA) acquisition of a two-way active avoidance task. Note that this task involves active and passive avoidance, as well as contextual conditioning; and that avoidance rather than freezing was selected for. As for the Carioca high-conditioned freezing rats, RLA rats display behavioral inhibition and a passive coping style (predominantly high freezing). As might be expected from the more complex paradigm, RLA also show enhanced sensitivity to threat, anxiety, fear, and frustration; and vulnerability to stress, which seem to link RLA rats to the internalizing and neuroticism domains of personality in humans.

Fernández-Teruel et al. (2023, p. 2) propose:

“From the [translational] perspective of the Research Domain Criteria framework such a profile suggests that RLA rats are mainly driven by ‘negative valence’. …They have enhanced sensitivity to threat/distress and behavioural inhibition, which is compatible with a high ‘neuroticism’ and low ‘extraversion’ personality profile …. Thus, RLA rats phenotypes seem to fall within the domain of ‘Internalizing’ spectra and ‘Fear’ and ‘Distress’ sub-factors in terms of [HiTOP].”

Fernández-Teruel et al. (2023)

According to them, the behaviorally inhibited and high threat-sensitivity profile of RLA rats is linked to “the enhanced activity of [hippocampus, medial prefrontal cortex and amygdala], together with more stress-responsive hypothalamus (HPA-axis) and bed nucleus of the stria terminalis” (Fernández-Teruel et al., 2023, p. 3).

In contrast, “the RHAs are behaviorally disinhibited, exhibiting a consistent low-anxious profile in unconditioned and conditioned tests of anxiety or fear. They are hyperactive and high exploratory, novelty/sensation seeking, impulsive, and present impairments of attention, sensorimotor gating (PPI), latent inhibition, startle habituation, working memory and cognitive flexibility” (Fernández-Teruel et al., 2023, p. 3). Due to these characteristics, the authors link RHA to an “externalizing” domain of human personality:

“Collectively, the above profiles are consistent with RHA rats being driven by ‘positive valence’ processes, and characterized by high reward sensitivity (and impulsivity) and impairments in the cognitive domain, which are compatible with a personality dominated by ‘Impulsivity/Sensation seeking’ traits … Hence, the RHA rats seem to fall within the ‘Disinhibited Externalizing’ spectra and ‘Substance Abuse’ sub-factor, and likely also within the ‘Thought disorder’ spectra; as they present various schizophrenia-linked traits, such as, e.g., hyperactivity, relative asociality, and impairments of inhibitory control (impulsivity) and attentional/cognitive processes.”

Fernández-Teruel et al. (2023, p. 4)

Associated with their “disinhibiting externalizing” profile, the brain of RHA rats shows immature features including hypo-frontality and disruption of the balance between excitation and inhibition. Hippocampal and amygdala function are also decreased while mesolimbic dopamine system tone is increased and there are changes in mGlu2, 5-HT2A, and GABA function. These data support the idea of “RLA rats as a valid model of anxiety/fear, stress, and frustration vulnerability, whereas RHA rats represent a promising translational model of neurodevelopmental alterations related to impulsivity, schizophrenia-relevant features, and comorbidity with drug addiction vulnerability” (Fernández-Teruel et al., 2023, p. 1).

Finally, the authors highlight the translational importance of the Roman lines, pointing out characteristics that are observed in these animals and human mental disorders: (1) differences in the volume of the hippocampus and amygdala are associated with behavioral inhibition; (2) impulsivity and novelty seeking are linked to differences in the striatum; (3) anxiety and motivated behaviors are correlated and might provide insights to frustration tolerance, drug abuse and emotional resilience; (4) predictive validity can be seen in the effects of anxiolytic/anxiogenic drugs; (5) cortical visual-evoked potentials function as a neurophysiological marker of disinhibitory behavior; (6) dopaminergic pathways in the striatum and in the prefrontal cortex are different in RHA and RLA rats and are associated with impulsive-sensation seeking; (7) alterations in serotonin 5-HT2A receptors density and its correlation to impulsive choice; (8) schizophrenic-like neurobiological alterations in the serotoninergic and glutamatergic pathways; and (9) schizophrenic-like behavior is associated with vulnerability to drug addiction and differences in the functional tone of the mesolimbic dopaminergic system.

### 2.4. The Maudsley model of emotionality

Blizard et al. (2023) aim at investigating the genetic background of Neuroticism using the Maudsley rat strains as a model. The Maudsley reactive (MR) and Maudsley nonreactive (MNR) rat strains were bidirectionally selectively bred (Broadhurst, 1960), respectively, for high and low open-field defecation (OFD) in the 1950s. Based on Calvin Hall’s work (Hall, 1934, 1951), OFD would be a measure of anxiety-like behavior and reactivity to threat, with face validity to humans in combat. Behind their behavioral selection, important genetic restriction occurred in relation to the “noradrenergic system where MNRs exhibit greater sustained cerulean response to chronic stress, and in the peripheral noradrenergic system where MNRs possess increased sympathetic tone in many organs” (Blizard et al., 2023, p. 3). This broad change in tone provides a basis for an attempt to show how alterations in the crosstalk between the central and the peripheral neuro-systems (CNS and PNS, respectively), using the irritable bowel syndrome (IBS) model that can be associated to neuroticism (once understood as “emotionality”), compares between the Maudsley rats and humans.

After describing evidence of disrupted norepinephrine (NE) pathways in the CNS (in particular the locus coeruleus) and the PNS (and its intestinal enervation) especially in response to stress in the MNRs, the authors establish the relationship between these alterations and the IBS syndrome in humans in regard to previously observed changes in NE receptors:

“We hypothesize that alterations in sympathetic tone, such as we have suggested to exist in the Maudsley model, could interact with these receptors to produce these functional disorders. Aside from presynaptic influences, the possibility exists that genetic selection for OFD may have also assorted different densities of colonic α_1_ receptors or different colonic receptor types in the two strains.”

Blizard et al. (2023, p. 4)

Part of the argument relies on the shared genetic background of IBS predisposition and anxiety in humans. This can be used to trace a parallel to the Maudsley rats, not so much in a causal manner as focusing on the correlation of the two processes. The trait tendency to anxiety-like behavior in rats has been used as a proxy for Neuroticism in humans (which can encompass pathological anxiety). This can be matched up with a correlation between Neuroticism and the risk of developing IBS and their shared heritability in humans:

“Bidirectional Mendelian randomization and other analyses also showed that anxiety or depression and IBS are the result of shared etiologic pathways rather than one causing the other. Applying the same logic to the Maudsley model it is possible that any influence of genetic selection on brain and behavior reflects the effect of the same genes that altered the peripheral sympathetic nervous system, not because one caused the other, as implied by the choice of the selection criterion, but because they are separate outcomes of the same neural pathways acting in the brain and the periphery. …”

After its initial conception and development, the Maudsley model became an object of fascination in its own right. More and more comparisons were made between the strains, each one appearing to add to the presumptive validity of the model. On the other hand, the findings were seldom held up as a window to elucidate the human dimension of Neuroticism. Obviously, disciplinary specialization made this difficult for both animal and human researchers. It is now time to use the model for its original purpose. In this brief review, we have tried to show that a bidirectional process of exchange between the animal model and the human dimension of Neuroticism can be productive. We have focused attention on the relationship between the respective roles of the central and peripheral nervous systems in emotional behavior in the animal model and raised questions about simplistic notions of cause and effect that can be fruitfully applied when considering the Neuroticism dimension. Seeing phenomena through a genetic lens also provides an excellent means of promoting animal/human exchanges, and this is facilitated by the extraordinary advances in understanding and analysis of the mammalian genome. This process of exchange needs to be strengthened.

Blizard et al. (2023, pp. 5, 7)

### 2.5. Rat models of attention deficit and hyperactivity

Both the CLF and RHA rats have unexpected impulsive and externalizing features (rather than being simple opposites of their anxiety/stress-linked counterparts). However, as yet, they are not used much to investigate impulsive behaviors. The field of hyperactivity and impulse-control disorders finds a more common model in spontaneously hypertensive rats (SHR). Originally selected for hypertension, the SHR genome acquired the distinct characteristics of altered sensitivity to delay of reinforcement, impulsivity, and inattention.

Wickens and Tripp (2024) review the evidence of altered dopaminergic mechanisms (in particular their dopamine transfer deficit/DTD hypothesis; Tripp & Wickens, 2008, 2009) in SHR rats. They highlight the strain’s importance as a tool for studying ADHD:

“Rodents with genetically determined behavioral characteristics provide opportunities for experimental study of brain mechanisms underlying those behavioral characteristics. Moreover, although they are also complex organisms, experimental animals can be bred selectively to express specific behavioral traits. Inbred strains provide homogeneity of genetic makeup, and cross-breeding can be used to determine if characteristics are genetically linked. Animal models also provide otherwise unattainable invasive and repeated measurements important for identifying underlying neural mechanisms. These have been particularly successful in the neurobiological investigation of mechanisms for positive reinforcement.”

Wickens and Tripp (2024)

As for other mental disorders (e.g., anxiety), ADHD could be viewed as an exaggeration of normal personality traits. However, Wickens and Tripp (2024) question its location within the antisocial subfactor of the HiToP model (Mullins-Sweatt et al., 2022) and propose to ground a dimensional conceptualization of ADHD in its neurobiological causes, despite this being beyond current human neurobiology. They invert their approach to a focus on the role of dopamine in the altered reinforcement sensitivity both in humans with ADHD and in the neurobiologically more tractable SHR line.

They provide a comprehensive description of how dopaminergic mechanisms respond to reward, leading to the DTD to explain the phenotype of ADHD. The key idea is that:

“…transfer of the dopamine signal from reward to reward-predicting cue ensures that dopamine release occurs at the right time to strengthen synaptic connections at the cellular level, even when the behavioral reinforcer is delayed. However, the success of this depends on the ability to learn the cue-reward association and complete the transfer of the dopamine signal to the cue. We have previously considered the possible consequences of failure to learn the cue-reward association. We refer to this as the dopamine transfer deficit (DTD) hypothesis (Tripp & Wickens, 2008, 2009).”

Wickens and Tripp (2024)

Altered timing of the phasic dopamine response from rewards to cues could be connected to the altered behavior of SHR. Besides changes in dopamine function, “such as lower basal dopamine levels, decreased release of dopamine and faster time course of dopamine clearance after release, the SHR also show higher sensitivity to delay of reinforcement than comparison strains, a stronger preference for immediate over delayed reward, … and differences in phasic dopamine release in response to reward and reward-predicting cues” (Wickens & Tripp, 2024).

The behavioral parallels between SHR and humans with ADHD include not only choice of small immediate rewards over larger delayed rewards in both choice delay and temporal discounting paradigms but also fitting core predictions of DTD, “steeper delay of reinforcement gradient, slower learning under partial reinforcement, faster extinction of learned behavior, a reduced partial reinforcement extinction effect, and increased sensitivity to individual instances of reinforcement” (Wickens & Tripp, 2024).

While ADHD does not fit clearly in the current models of personality psychopathology, Wickens and Tripp (2024) argue that the variations of dopamine response to reward and reward predictive cues could not only explain the continuum characteristics of ADHD but also traits underlying healthy personality dimensions. In particular, they explore the association of DTD with impulsivity, distractibility, and persistence; and its potential link to conscientiousness, agreeableness, and neuroticism. Therefore, Wickens and Tripp (2024) make their case on the importance of studying the DTD theory in SHR as a model for not only better understanding ADHD but also refining “personality dimensions by inclusion of neurobiological variations in reinforcement mechanisms.” Thus, it may be “that one or more relevant dimensions are missing from the personality model. The DTD hypothesis suggests some facets of those dimensions.”

### 2.6. Implications of the models for human trait analysis

In the introductory paper of this special issue (Lages & McNaughton, 2022), we made an argument of how the genetic background and the neurobiological constitution of a being could interact with the environment to define personality traits. In the current review, we see how this happens in practice using four rat models of mental disorders that act as an exacerbation of these traits. Three of the pairs of lines (the Cariocas, the Romans, and the Maudsleys) were produced by selective breeding based on behavioral responses to threatening environment. The outcome was two bidirectional opposed groups representing extreme behaviors. The fourth line, SHR, was generated based on the wish to obtain a model of high blood pressure, but ended up producing animals with altered attention and motor-activity. Importantly, with the selective breeding, all rats within each line share a similar genetic background that can be associated to their distinctive behavioral pattern. Mediating between genome and behavior are neurophysiological alterations.

In the CHF and RLA rats, amygdala, hippocampus, and glutamatergic and serotonergic pathways are altered, enabling their highly passive-defensive behavior, and altering their response to anxiolytic drugs (Cruz et al., 2024; Fernández-Teruel et al., 2023). These same circuits are altered comparably in anxiety and fear disorders in humans (Shin & Liberzon, 2010; Tovote et al., 2015). Fernández-Teruel et al. (2023) extend this perspective and frame the RLA rats within the domain of “Internalizing” spectra of HiTOP (Michelini et al., 2021). Similarly, based on the genetic background and neurophysiological alterations of the Maudsley Reactive rats, in particular the disrupted norepinephrine (NE) pathways in the CNS and the PNS, Blizard et al. (2023) trace a parallel between this model and Neuroticism in humans, rather than a cause-consequence relationship, the concomitant heritability of anxiety traces, alterations in bowel functioning due to stress, and neuroticism happen in both humans and rat lines, making the comparison between them, plausible.

On the other side are the lines with dampened defensive behavioral responses. Evidence described previously with CLF, RHA, MNR, and SHR rats show alterations in the reward machinery and dopaminergic pathways, which suggests them as prospective models of attention deficits and hyperactive disorders given that similar deficits are seen in humans (Bonvicini et al., 2016; Del Campo et al., 2011). In the view of Blizard et al. (2023), hyperactivity, relative asociality, and impairments of inhibitory control (impulsivity) and attentional/cognitive processes place these animals as proxy of “disinhibited externalizing” spectra of human personality.

Thus, we have tried to show that anchored on stem genetic and neurobiological characteristics of both humans and rat lines, key mechanistic aspects of human personality traits (often in their disrupted presentation as mental disorders) can be studied with rodent strains. In the next section, we take our final step, diving deeper in the most primitive circuits governing behavior and their conserved parallels to human personality.

## 3. Conservation – personality in fish

### 3.1. Reduced models of conserved trait elements

A key element of the idea of conserved characteristics is that in simple organisms, it will be easier to see the fundamentals that arose in a shared common ancestor. However, the simpler organism will have experienced as many million years of divergent evolution as have humans – so both commonalities and differences should be instructive. We must look for homologies not identities.

That said, to a large degree, we can see the steps we have taken from primate to rat to fish as progressive stripping of layers of cortical perceptual and cognitive complication to reveal a primordial adaptive emotional and motivational machine. If fish do not have “personality” in some sense that does not mean they cannot instruct us about mechanisms that underlie our own. Certainly, they can instruct us about elements of emotion and its psychopathology (de Abreu et al., 2020; Fontana et al., 2019; Jesuthasan, 2012; Loonen & Ivanova, 2015, 2016; Mathuru & Jesuthasan, 2013). The next two sections support the idea that, in addition, fish do have “personality” elements many of which can be mapped back to rats and humans.

### 3.2. A 5-dimensional trait overview

Luchiari and Maximino (2023) provide an overview and critique of what is known about fish personality. Their discussion includes “metatheoretical issues, personality dimensions, and applications to neuroscience and psychopathology.” These two applications are considered further in sections 4 and 5. Important metatheoretical issues (largely shared with human personality research) are as follows: How one defines personality (particularly in relation to non-linguistic species); how different dimensions are measured; what unit of analysis should be used; how to best approach the identification of dimensions (i.e., through a bottom-up or a top-down approach, or an iterative combination of both); and how far one should be concerned about mechanisms. Their key ultimate conclusion is that fish can be used to understand the evolutionary basis of personality, but with conserved systems showing a degree of homology rather than a complete identity.

They see fish personality as classically divided into five dimensions: shyness-boldness; exploration-avoidance; activity; aggressiveness; and sociability. However, they note that some caution is needed in seeing these as the basis of individual differences since most work in this area assesses differences between distinct populations (see also the rat work described in section 2), with little analysis of within-individual correlations. Critically, in at least one case (Lee & Bereijikian, 2007), “a specific stability of correlations between behavioral variables at the population level did not hold at the individual level” (Luchiari & Maximino, 2023, p. 2). They emphasize (p. 3) that “simply referring to the term used for a specific dimension is not enough, and grasping the full meaning of that dimension/trait is only possible by considering the context and methods of each study.” We would see this labeling problem as identical to the problem with scale labels versus actual item content with questionnaires and so particularly important (for both ends) when attempting translation. Indeed, this is one area where attempts at translation may clarify the nature of the conventional human traits as a result of the required functional analysis.

Their Table 1 details assessment of these dimensions as: (1) shyness-boldness via freezing, avoidance, and suppression of feeding by direct threats; (2) exploration-avoidance via a variety of responses to potential threat (e.g., emergence, approach to novelty); (3) activity via speed and distance covered in a variety of tests; (4) aggressiveness via display to a mirror image or conspecific; and (5) sociability via time and distance from conspecifics (and time inspecting a predator when a conspecific is present). The measures used (but not necessarily the trait structures) show clear homology with functionally related rat behavior.

With 5 fish dimensions, it is also tempting to ask how far these can map to the domains of the human Big 5 (Costa & McCrae, 1985, 1995; McCrae & Costa, 2008). However, Luchiari and Maximino (2023, p. 8) say:

“…to the best of our knowledge, no attempts have been made to map fish data and the Big Five model; it could be argued that shyness-boldness could conceptually map to Neuroticism, exploration-avoidance could conceptually map to Agreeableness, activity to Conscientiousness, aggressiveness to Extraversion, and sociability to Openness/ Intellect. But it could be similarly argued that sociability should map to Extraversion, for example. The difficulty, again, lies at the metatheoretical level and can be solved partially by addressing that level in conjunction with further empirical research.”

They (p. 8; see also Maximino et al., 2012) see an approximation of fish personality to other animals, including humans, being more likely to be achieved via Reinforcement Sensitivity Theory:

“RST, which has been developed from Jeffrey Gray’s [(Gray & McNaughton, 2000)] neuropsychology of anxiety (Corr, 2002, 2004; Corr & Perkins, 2006). RST views significant affective events as either positive or negative, postulating three interacting systems that process these events and control behavioral responses to them: The fight-flight-freeze system (FFFS), which mediates reactions to all aversive stimuli, with the associated emotion of fear; the behavioral approach system (BAS), which mediates reactions to all appetitive stimuli, with the associated emotion of anticipatory pleasure; and the behavioral inhibition system, responsible to solve conflicts between approach (BAS) and avoidance (FFFS).”

This motivational approach (and its mapping to neural systems) should work well for shyness-boldness and exploration-avoidance (particularly given the homology of the fish behaviors assessed with those in the rat data from which RST was developed). However, as with the limited range of RST constructs within the human Big 5 system, the other dimensions require more careful analysis. Thus, while RST (with its highly conserved neural systems) appears to be the most promising case of fish-rodent-primate-human trait mapping, it is clearly only partial, but nonetheless a convenient anchor for future more extensive work.

### 3.3. Trait sensitivity and cognitive bias

Buenhombre et al. (2024) provide a more targeted picture of the relation, in fish, between personality traits, cognitive bias processes (CBP), and stress resilience (including psychopathology). They start (p. 1) from the same fundamental position as Luchiari and Maximino (2023):

“…the functional homology of neural regions in fish is well-conserved in rodents, and their behaviour exhibits sufficient complexity to enable translation to both rodents and humans (Khan & Echevarria, 2017). As such, biological traits that are similar between fish and mammals have been widely utilized in models of anxiety and stress neurobiology. … In fish as in other species … coping with stress and cognition are closely related processes. For instance, a fish’s appraisal of stimuli, rather than the intrinsic characteristic of the stimuli, can have significant effects on stress responses and related emotion-like or affective states. … Two primary sources of variation in CBP have been studied: the living environment and personality traits (Kremer et al., 2021) … [which] may interact to affect CBP. … [One can then] categorize individuals into two phenotypic traits: those with a stable [positive cognitive bias], referred to as ‘optimistic’, and those with a stable [negative cognitive bias], referred to as ‘pessimistic’. This categorization has played a pivotal role in exploring the idea that CBP could be a trait contributing to the development, persistence, and recurrence of stress-related disorders such as depression and anxiety (Noworyta-Sokolowska et al., 2019; Noworyta et al., 2021).”

On this view, fish and other vertebrates respond to environmental stimuli according to their current emotional state showing attention bias (towards specific stimuli), judgment bias (in assessment of ambiguous cues), and sensitivity to reward shift (sensitivity to *both* negative and positive feedback reflected in, e.g., “loss aversion” in humans). These CBP can result from both states and stable and enduring behavioral traits.

Buenhombre et al. (2024) focus on how animals placed in two opposing stable cognitive bias categories (positive or optimistic, and negative or pessimistic) differ on the development, persistence, and recurrence of stress-related disorders such as depression and anxiety. Their hypothesis is that CBP in some species may incorporate aspects of stable personality traits and more transient affective states, similar to CBP in humans, and, therefore, it might interact with various personality traits, influencing stress resilience or vulnerability in animals. The authors highlight that due to the influence of individual differences in CBP, such as age, sex, personality, and strain, owing to environmental and genetic variations, along with their interplay, researchers willing to study the impacts of CBP in stress-elicited responses must consider these aspects and in particular the possibility of stable (personality) traits in fish.

Importantly, the externally observable behavioral differences map to similar serotonergic and hypothalamo-pituitary-adrenal variation as in mammals (see also section 4). Not only, then, may fish provide models of traits that are conserved in humans but “insights gained from fish research on CBP may contribute to cognitive models suggesting that stress-related disorders in humans are linked to biases in cognitive processing (Beck, 2008)” (Buenhombre et al., 2024).

## 4. Attributing “personality” to non-human animals

In what we have covered so far, the term “personality” has been used quite loosely, and it has been taken as given that the non-human data on conserved systems provide, at the least, useful information about mechanisms that can contribute to human personality (see also McNaughton & Corr, 2022; McNaughton & Smillie, 2018). That is, if “personality is an abstraction used to explain consistency and coherency in an individual’s pattern of affects, cognitions, desires and behaviors. … [And, if] the task of the personality researcher is to identify the consistencies and differences within and between individuals … *and … to explain them*” (Revelle, 2007, p. 37, our emphasis), then it is the task of the research included in this special issue to provide fundamental explanations.

But, how far such data provide explanations of personality itself as opposed to explanations of simple contributing processes raises a metatheoretical question “which animals have personality?” (Adolphs & Xu, 2024, title):

“Human personality generally refers to coherent individuating patterns in affect, behavior, and cognition. We can only observe and measure behavior, from which we then infer personality and other psychological processes (affect, cognition, etc.). We emphasize that the study of personality always explains or summarizes patterns not only in behavior but also in these other psychological processes inferred from behavior. We thus argue that personality should be attributed only to nonhuman animals with behaviors from which we can infer a sufficiently rich set of psychological processes. The mere inference of a biological trait that explains behavioral variability, on our view, is not sufficient to count as a personality construct and should be given a different term. Methodologically, inferring personality in nonhuman animals entails challenges in characterizing ecologically valid behaviors, doing so across rich and varied environments, and collecting enough data. We suggest that studies should gradually accumulate such corpora of data on a species through well-curated shared databases. A mixture of approaches should include both top-down fit with extant human personality theories (such as the Big Five) as well as bottom-up discovery of species-specific personality dimensions. Adopting the above framework will help us to build a comparative psychology and will provide the most informative models also for understanding human personality, its evolution, and its disorders.”

This view can be rephrased:

“…personality needs to be inferred when we want to explain behavioral patterns so complex that we need to infer psychological terms: these behavioral patterns cannot be explained efficiently by biology alone…. To be sure, we would see all psychological processes ultimately as biological (and, for that matter, chemical and physical) but these disciplines have different terms, different explanatory aims, and offer different efficiency in their explanation. [But] stable differences only in running speed, reaction time, a limp on one side, or a tremor … do not need psychological explanations. [Likewise,] individual differences in memory or attention alone are just that – explanation with a personality trait should be reserved for those cases that would be incomplete otherwise. …

No doubt the view described above will be persuasive in attributing personality to only some species (like humans, monkeys, dogs, and probably rodents), exclude it from others (like sea anemones), and leave a lot in a gray zone where debate and further studies are needed (like fish or octopuses).”

Adolphs and Xu (2024)

Adolphs and Xu (2024) suggest a range of criteria from which one can infer non-human personality and emphasize the importance of larger datasets, consensus ethologically based coding, and the use of naturalistic settings – with special attention being paid to context dependency. That is when specific behaviors vary with context, if an inferred construct is stable this can be taken ans relating to personality. They also see a role for the application of extant human personality theories albeit with the dangers of overfitting the data to expectations and of anthropomorphizing. They suggest that a solution, here, is to use data-driven approaches to animal personality, allowing species-specific dimensions to be extracted as well as similar approaches to human social media and smartphone usage. They also argue that both top-down and bottom-up approaches should be used in tandem.

## 5. The brain – an anchor for personality and psychopathology

Color vision, whiskering, and the lateral line system will give primates, rats, and fish a very different picture of the world in sensory and cognitive terms. However, whatever the sensory source, detection of danger (e.g., a predator) will activate the same subcortical systems across these species and, if this includes the periaqueductal gray, generate panic. Likewise, anticipation of danger and a resultant conflict between approach and avoidance will release stress hormones, with cortisol and corticosterone producing similar reactions in humans and rats/fish, respectively.

The human cortex may allow mental time travel further into the future and so alter what stimuli elicit any particular emotion. But trait variation in emotional sensitivity is independent of the specific stimulus used to elicit the emotion and selection for responses such as defecation and avoidance that humans show in response to threat produces appropriate trait differences in rats that can be linked to neural systems also implicated in the human responses (§2). Likewise, variation in autism-related behavior in primates is associated with what in humans is seen as the social brain network (§1.4).

In the same way that the effective stimuli may vary from species to species (and from individual to individual within a species), the form of the resultant adaptive responses can vary. While both rats and humans can show defensive attack, humans do not normally leap at the face of a predator and try and bite its nose. But the species-specific behaviors that result from activating a particular brain structure (in this case the medial hypothalamus) share a function (in this case “fight” elicited from a species-general fight or flight system) that can be seen as the basis for homology of the trait across the species.

Especially with the use of drugs, it is relatively easy to map psychopathologies to brain structures. At this point, the link to traits can be made if we see both healthy and pathological behavior as organized in terms of trait hierarchies. Importantly, both pathology and change in species may affect the degree of intercorrelation of facets and so the apparent trait structure at the level of, e.g., dominance (§1) without any fundamental change at the facet level. This is similar to arguments for seeing RST as easier to translate among species than, e.g., the Big 5 as a whole.

This special issue focuses most on the suitability of different species for different aspects of personality research. This is not to ignore the importance of the brain (which has been important for many of the contributions). Rather it is to see what is known about the brain and states as a first step for many homologies – and to see an integrated comparative neural overview as a task for the future.

## 6. Conclusions

This special issue has mapped some of the main lines of investigation of human personality in non-humans. The contributions have been grounded in evolution, with implicit and explicit arguments that phylogenetic conservation of many neural structures allows translational research into mechanisms of personality traits and, with species-specific caveats, personality structure. We have sampled phylogeny at 3 levels: primates, rats, and fish. In each of these cases, work has been done that plainly connects with human personality from a healthy and/or pathological perspective.

### 6.1. The fundamental fish

In the simplest models described in the special issue, individual and group differences in fish are observed in response to environmental changes (most commonly, stressful events). Individual differences in response to fixed environmental changes suggest primitive traits of personality and have led to the development of behavioral measures to categorize them. One important conclusion from this work is that fish can be seen not only to have personality but also to demonstrate traits that can be related to human personality, if only at the facet level. A second important conclusion is that with such traits (as with other aspects of psychology) finding a homology is definitively not demonstrating an identity. Indeed, it can be argued that the variation in the precise form, and structure, of traits with homology based on common function and neural conservation is where inter-species comparisons can be particularly instructive. For example, fish may have a 5-dimensional trait structure but this is hard to map to the human Big 5 and may be easier to fit with the motivational systems of RST (Luchiari & Maximino, 2023, see §3.2). At the more detailed level, fish appear to be similar to humans in showing cognitive bias in the appraisal of stimuli and similar stress-related reactions and resilience (or otherwise) to them (Buenhombre et al., 2024, §3.3). Again, the homology seems clearest in terms of basic (and so conserved) emotional reactions rather than higher-order social ones. Although they have evolved over similar period of time to our lineage, comparison with fish provides us with the clearest picture of the common conserved systems that still provide a foundation for our personalities.

### 6.2. The selected rat

Rat strains are easy to breed and maintain in the laboratory. They also come with a wealth of behavioral, neural, biochemical, physiological, and pharmacological data providing a range of disease models and tests for pharmacotherapeutic action. As a result, the bulk of the rat personality studies in the special issue involve bidirectional selection that results in “high” strains that are seen as models of psychiatric disorder or its risk factors. Interestingly, the “low” strains can often also be viewed as models of psychiatric disorders, or related personality dimensions, that are not simple opposites of the “high.” Selection has often then delivered two models for the price of one. In a similar fashion, even unidirectional selection (for spontaneous hypertension) can deliver an additional model (of ADHD).

This work has two features that are important for the study of human personality and psychopathology. First, each rat model of a psychopathology allows broad investigation of behaviors and physical responses that arguably represent extremes of a trait in the original pre-selection population, where this extreme also represents an exaggeration of human personality dimensions. This argues for related trait hierarchies of healthy and pathological personality (Kotov et al., 2021; Michelini et al., 2021). Second, and particularly importantly, these response patterns can be traced to key, primitive, brain circuits that can bolster their homology with primates, including humans, tested with techniques such as MRI.

Somewhat different rat selection criteria have delivered “high” strain models of trait anxiety (Cruz et al., 2024, with characteristics reminiscent of generalize anxiety disorder, §2.2); internalizing more generally (Fernández-Teruel et al., 2023, §2.3), and, yet more generally, neuroticism/emotionality (Blizard et al., 2023, who demonstrate interesting links to irritable bowel syndrome, §2.4). The equivalent “low” strains seem to model impulsivity and externalizing to some extent but, interestingly, it is selection for high blood pressure that has delivered the most generally accepted model of ADHD and provided evidence for the hypothesis that this depends on a dopamine transfer deficit (Wickens & Tripp, 2024, §2.5). These models make clear that adaptive selection can operate at any level of the current proposed personality hierarchies and also show how a disorder such as ADHD may not fit neatly within them (Wickens & Tripp, 2024, §2.5) nor do they fit neatly with RDoC (Michelini et al., 2021).

### 6.3. The social primate

If we want to understand aspects of personality linked to social perception or action then primates in general and apes in particular offer the best examples of social groups similar to humans. It is no surprise then that the primate contributions to the special issue focus on social communication (and its disorder) and do so from a strong translation perspective.

Thus, Wilson and Masilkova (2023, §1.2) start with the hypothesis that *human* male relative facial width is linked to dominance and then test it in *non-humans*. They argue from the primate data that discrepancies in human research would be resolved with more broad ranging and nuanced analysis. Consistent with this, Altschul (2024, §1.3) treats dominance as multifaceted, being essentially similar across primates but with a drift of its facets during hominid evolution so that they now each align more with separate traits than with a single dominance factor. Somewhat reminiscent of the rat work with artificially selected strains, Hopkins et al. (2023, §1.4) analyzed natural variation in attention skills (using a similar task to human testing) in a large (N = 189) cohort of chimpanzees (*Pan troglodytes*) and concluded that low versus high scorers provide “an excellent model for understanding the mechanisms underlying social impairment related to [Autism Spectrum Disorder].” Critically, they obtained Big 5 personality scores from carer ratings and gray matter volume from MRI – finding involvement of extraversion and of the social brain network.

### 6.4. Onward to comparative personality neuroscience

As noted in §5, we see this special issue as providing, for personality neuroscience, equivalent reasons for research on non-humans as those that have driven other areas of neuroscience and psychology. In particular, both in terms of use of appropriate models and of use of the comparative method generally, we think it is important both to know what models are available and to urge that the species be compared and their data integrated.

In doing this, one must take great care at the metatheoretical level to be clear as to what personality is, how it should be measured, and what form its neural basis could take. The importance of metatheory is emphasized by Luchiari and Maximino (2023, §3.2) in the attribution of traits to fish and, in particular, any attempt to link fish personality structure to human. Adolphs and Xu (2024) ask “which animals have personality”? They emphasize the importance of the complexity of behavioral reactions for the need to attribute personality being doubtful of fish and (with a caveat) excluding sea anemones.

We believe that progress in human personality research must point toward looking to the stem neural mechanisms in different species and involve a revision of epistemological and metatheoretical issues that has been long overlooked. Importantly, for true comparative work, one must make allowance for the disparate features of species while attempting to study the commonalities (both their conservation and their divergence). Using visual stimuli of threat, for example, is clearly a mistake if the species is blind but would react briskly to an auditory or olfactory stimulus. Also important, here, is to avoid the lexical trap. Yes, one can use observer reports to assess primates with Big 5 questionnaires – but that is a very special case. It is worth, then, asking the different species the same question (from a paradigmatic point of view). It is easy to see how to test a rat like a fish for its defensive reactions. Luckily, we can now use virtual predators (that can genuinely hurt the participant when they catch an avatar) to generate fish/rat-like defensive reactions while studying the human participant’s brain with fMRI and obtain rat-predicted neural reactions (Bach et al., 2014; Korn & Bach, 2019; Qi et al., 2018) that depend on trait anxiety (Fung et al., 2019).

It may be simplest to start such truly comparative work with RST that is based on an already comparative neuropsychological theory (updated by McNaughton & Gray, 2024) and has clear links to the fish and rat work. However, a clear start has already been made on the Big 5 and its links to neural systems with primates (Hopkins et al., 2023, §1.4; Weiss, 2017, 2022).
